# Feasibility of a novel one-stop ISET device to capture CTCs and its clinical application

**DOI:** 10.18632/oncotarget.13823

**Published:** 2016-12-08

**Authors:** Fangfang Chen, Shuyi Wang, Yuan Fang, Liang Zheng, Xuan Zhi, Boran Cheng, Yuanyuan Chen, Chunxiao Zhang, Dongdong Shi, Haibin Song, Congli Cai, Pengfei Zhou, Bin Xiong

**Affiliations:** ^1^ Department of Oncology, Zhongnan Hospital of Wuhan University, Hubei Cancer Clinical Study Center, Hubei Key Laboratory of Tumor Biological Behaviors, Wuhan, China; ^2^ Department of Circulating Tumor Cells, YZY Medical Technological Company, Wuhan, China

**Keywords:** circulating tumor cells (CTCs), clusters of circulating tumor cells (CTC-clusters), epithelial-mesenchymal transition (EMT), isolation method by size of epithelial tumor cells (ISET), colorectal cancer (CRC)

## Abstract

**Introduction:**

Circulating tumor cells (CTCs) play a crucial role in cancer metastasis. In this study, we introduced a novel isolation method by size of epithelial tumor cells (ISET) device with automatic isolation and staining procedure, named one-stop ISET (^os^ISET) and validated its feasibility to capture CTCs from cancer patients. Moreover, we aim to investigate the correlation between clinicopathologic features and CTCs in colorectal cancer (CRC) in order to explore its clinical application.

**Results:**

The capture efficiency ranged from 80.3% to 88% with tumor cells spiked into medium while 67% to 78.3% with tumor cells spiked into healthy donors’ blood. In detection blood samples of 72 CRC patients, CTCs and clusters of circulating tumor cells (CTC-clusters) were detected with a positive rate of 52.8% (38/72) and 18.1% (13/72) respectively. Moreover, CTC positive rate was associated with factors of lymphatic or venous invasion, tumor depth, lymph node metastasis and TNM stage in CRC patients (*p* < 0.01). Lymphocyte count and neutrophil to lymphocyte ratio (NLR) were significantly different between CTC positive and negative groups (*p* < 0.01).

**Materials and Methods:**

The capture efficiency of the device was tested by spiking cancer cells (MCF-7, A549, SW480, Hela) into medium or blood samples of healthy donors. Blood samples of 72 CRC patients were detected by ^os^ISET device. The clinicopathologic characteristics of 72 CRC patients were collected and the association with CTC positive rate or CTC count were analyzed.

**Conclusions:**

Our ^os^ISET device was feasible to capture and identify CTCs and CTC-clusters from cancer patients. In addition, our device holds a potential for application in cancer management.

## INTRODUCTION

Tumor metastasis is the main cause of cancer-related death. Circulating tumor cells (CTCs) play a crucial role during this malignant progression [1]. In recent years, CTCs have been considered as ‘liquid biopsy’, as they could supply important information of primary tumor and distant metastasis for clinical practice. The presence of CTCs also has been validated to associate with worse prognosis in many cancer types, including breast, prostate and colorectal cancer [2–4]. Besides, CTCs hold great potential in cancer management, such as monitoring treatment response and performing personalized therapy [5]. However, owing to the extremely low abundance of CTCs [6] (one tumor cell in millions of blood cells), detecting and characterization of CTCs has still been technically challenging.

Recent studies of CTCs are mainly based on the use of CellSearch^®^ [2–4, 7], microfluidic devices [8–10] or magnetic separation methods [11, 12]. These methods were all dependent on epithelial cell markers, such as epithelial cell adhesion molecule (EpCAM). Although these methods exhibited great potential in isolation and identification of CTCs, many recent studies have validated that these methods may miss important subgroup of CTC and clusters of circulating tumor cells (CTC-clusters). Because CTCs and CTC-clusters may undergo the process of epithelial-mesenchymal transition (EMT) which increase the capacity of invasiveness, immune escape and metastasis [13, 14]. During this process, CTCs and CTC clusters acquire mesenchymal phenotype and loss of epithelial features such as reduced or no expression of EpCAM and/or CK [14, 15]. Therefore, these methods based on epithelial markers were not feasible to detect tumor cells with EMT features. Although some methods based on negative enrichment principle or combined antibodies against CTCs have improved the capture efficiency [16–18], the disadvantages of expensiveness and low through-put limit their clinical application. Meanwhile, these methods usually use immunofluorescence (IF) staining method to identify CTCs, which is recently considered a standard method. However, there are still some disadvantages. Such as, it takes a series steps which cost much time and money and only reveals the expression of specific marker.

Isolation by size of epithelial tumor cells (ISET) technique is widely used in CTCs detection [19–21]. Tumor cells are isolated and retained by a membrane filter because of their larger size despite of the expression of markers. In recent studies, ISET device was demonstrated to be more sensitive than CellSearch system in detecting CTCs from cancer patients [20, 22]. Additionally, ISET device has revealed significant advantage of capture CTC clusters over CellSearch system [23–25]. Thus, ISET devices have their own advantages and hold great potential to isolation and identification of CTCs from cancer patients. However, most present ISET devices are operated by hands [20, 21, 26] and often utilize IF staining for identification. Nowadays, the cellular morphology method also has been widely used to identify CTCs and the cytomorphological criteria has been proposed by other research groups [27–28]. The staining method could show the morphology of the tumor cells despite their heterogeneity in expression of molecular makers. Wright's staining as one method of cellular morphology staining, has advantages of simple and convenient that it only takes a few minutes while IF staining usually takes more than 10 hours. Thus, utilization of ISET device combined with Wright's staining hold the potential of one-stop capture and identification for CTCs and a promising application in clinical practice.

Colorectal cancer (CRC) is the third most common cancer and the fourth most common cancer cause of death globally, accounting 1.2 million new cases and 600000 deaths per year [29]. Many studies have demonstrated that the CTC count at baseline was an independent prognostic factor for progression free survival (PFS) and overall survival (OS) outcomes in CRC patients [30–32], and the change of CTCs status during treatment was significantly associated with tumor response in CRC patients receiving chemotherapy [33–35] or target therapy [36]. The precise detection of CTCs may be a powerful tool in CRC for early diagnosis, prognosis prediction, personalized therapies and cancer surveillance [37]. However, there were little studies about detecting CTCs from CRC patients by ISET device plus Wright's staining, and even less studies on further exploration of clinical application of ISET device in CRC.

Herein, we fabricated a novel ISET device named one-stop ISET (^os^ISET), with automated procedure of isolating and Wright's staining. In this device, the isolation is carried out using a polymer membrane made by biocompatible parylene and with programmed Wright's staining on retained cells for identification. The ^os^ISET device is improved in the automatic isolation and staining procedure without human intervention and could finish the whole procedure within 10 minutes with advantage of high-throughput. In this study, we focus on three aspects of ^os^ISET: 1. The capture efficiency of ^os^ISET device and the concordance of IF staining and Wright's staining in identification CTCs. 2. The feasibility for detecting CTCs from cancer patients by ^os^ISET. 3. The clinical value of CTCs and potential use for ^os^ISET device in CRC patients.

## RESULTS

### Capture efficiency of the device

**Figure 1:**
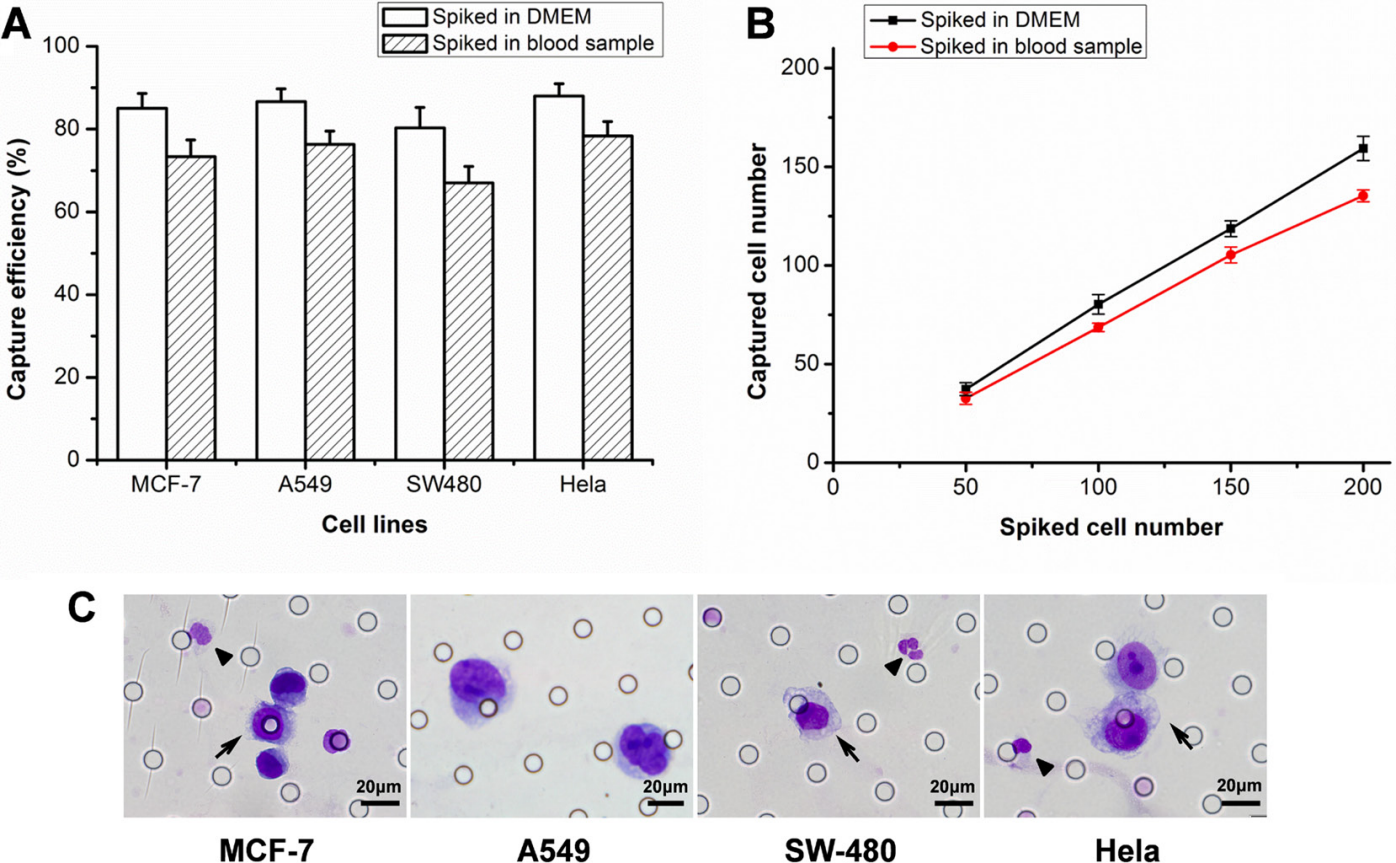
The results of capture efficiency tests (**A**) The capture efficiencies of different tumor cells sipked into DMEM or blood samples. (**B**) Captured SW480 cell number against the number of spiked in DMEM or blood samples at different concentrations. The error bars represent a mean ± standard deviation from three repeats. (**C**) Wright's staining of captured MCF-7, A549, SW480 and Hela cells. The arrows indicated tumor cells and the triangle (▲) indicated white blood cells (WBCs).

The cell lines selection was base on the consideration of including both EpCAM positive cells (human breast cancer cell MCF-7, human lung cancer cell A549, human colonal cancer cell SW480) and EpCAM negative cells (human cervical caner cell Hela). The capture efficiencies of detecting spiked A549, MCF-7, SW480, Hela cells into DMEM were 85%, 86.7%, 80.3% and 88% respectively. When spiked into blood sample, the capture efficiencies were 73.3%, 76.3%, 67% and 78.3% respectively (Figure 1A). In detecting spiked SW480 cells from DMEM at concentrations of 50, 100, 150, 200 cells per 2.5 mL, the capture efficiency was 74.7%, 80.3%, 79.1%, 79.7% respectively. The capture efficiency was 65.3%, 68.7%, 70.2%, 71.3% when detecting SW480 cells from healthy donors’ blood sample at above concentrations (Figure 1B). Figure 1C showed spiked tumor cells retained and stained by ^os^ISET device.

### The concordance of IF staining and Wright's staining

**Figure 2:**
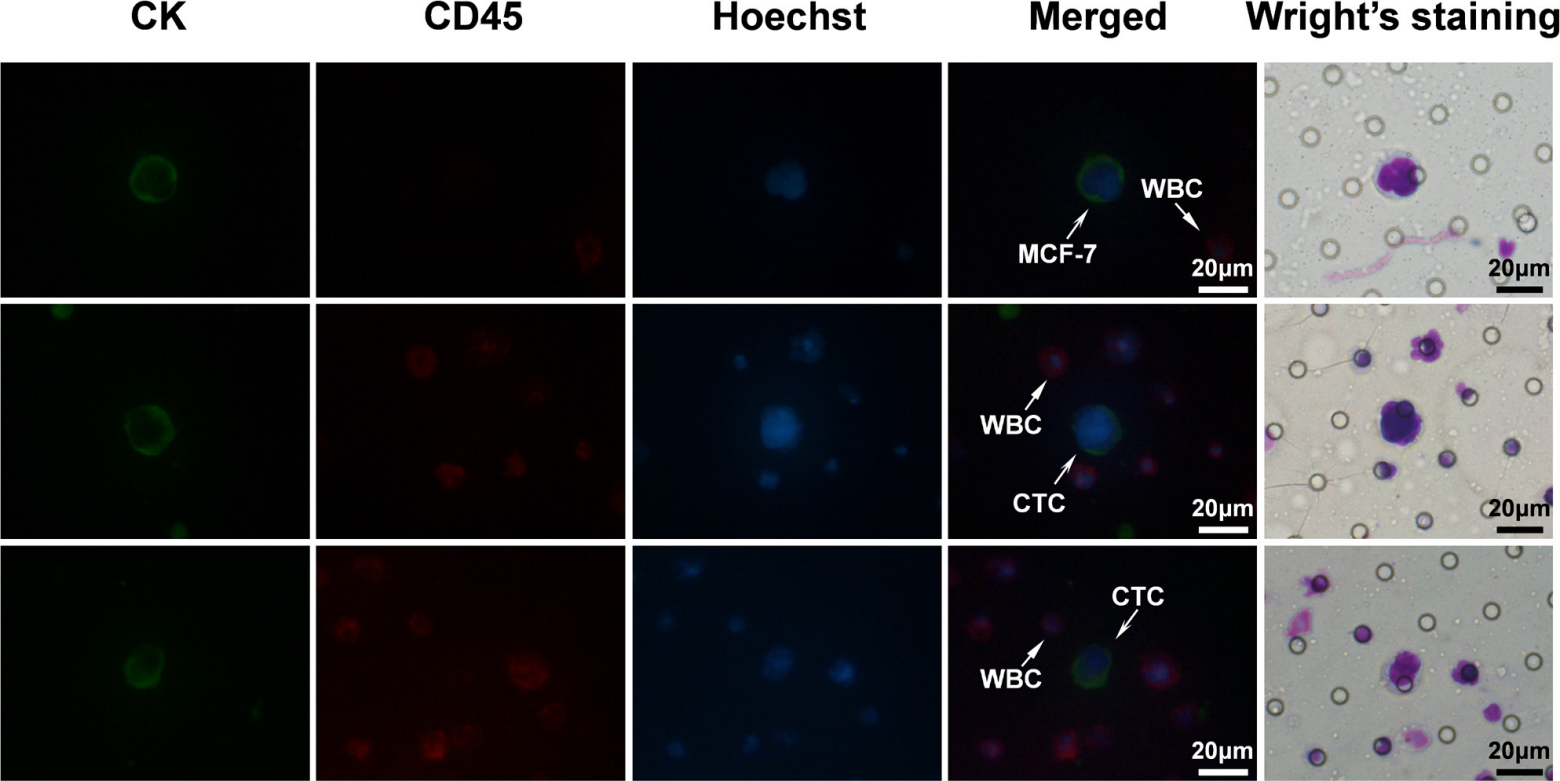
The images of IF staining and Wright's staining for the same samples MCF-7 tumor cells spiked into healthy blood samples were set as positive control. CK+/CD45-/Hoechst+ cell was scored as CTCs and CK-/CD45+/Hoechst+ cell as WBCs.

Figure 2 showed the two staining methods had identified the same CTCs according to respective criterion either in spiked blood sample or patient samples. Thus, Wright's staining could be a feasible way to identify CTCs.

### Detecting results of clinical samples

**Figure 3:**
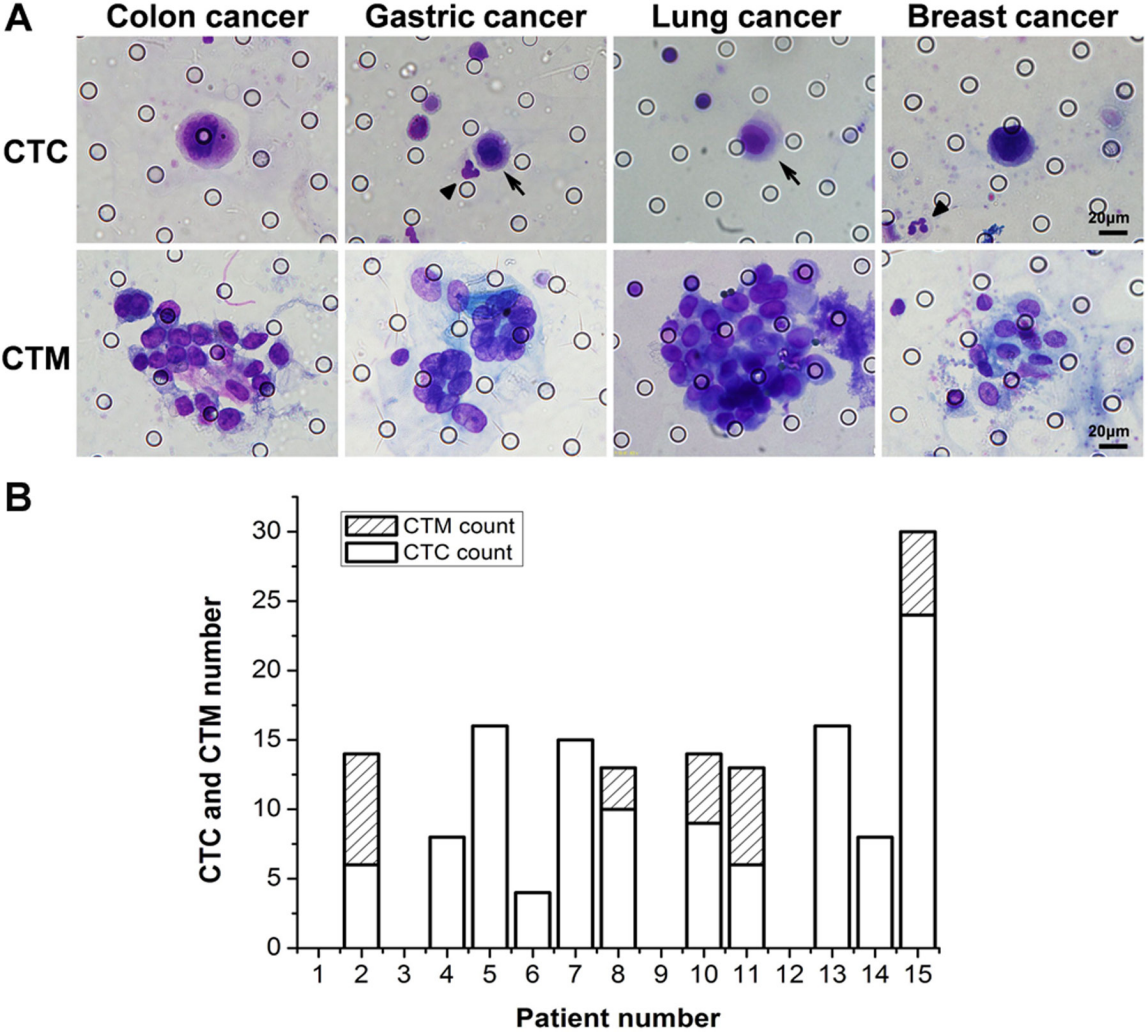
The detection results of blood samples from 15 cancer patients (**A**) Images of Wright's staining for isolated CTCs and CTC clusters from cancer patients. (**B**) The number of captured CTCs and CTC clusters in blood samples from 15 cancer patients. The arrows indicated CTCs and the triangle (▲) indicated WBCs.

To validate clinical feasibility of ^os^ISET, we collected blood samples from cancer patients, including 5 breast cancer, 5 lung cancer, 5 gastric cancer. The clinical information of cancer patients were collected in Table 1. CTCs and CTC clusters were detected from these clinical samples. In our study, the cellular morphology and cell nucleus were showed clearly by Wright's staining (Figure 3A). Enumeration results were summarized in Figure 3B. CTCs were detected from 11 patients and the CTC count ranged from 0–24, while CTC clusters were found in 5 patients with number ranged from 0–8. No CTCs or CTC clusters were found in blood samples of 25 healthy donors.

**Table 1:** CTC and CTM counts of 15 cancer patients detected by ^os^ISET device

Cancer species	Patient	CTC count	CTM count	TNM stage	Metastatic
Breast cancer	1	0	0	T1N0M0	−
	2	6	8	T2N0M0	−
	3	0	0	T2N1M0	−
	4	8	0	T2N2M1	+
	5	16	0	T4NXM1	+
Lung cancer	6	4	0	T2N0M0	−
	7	15	0	T2N1M0	−
	8	10	3	T2N2M0	−
	9	0	0	T3N1M0	−
	10	9	5	T2NXM1	+
Gastric cancer	11	6	7	T2N0M0	−
	12	0	0	T3N0M0	−
	13	16	0	T3N2M0	−
	14	8	0	T4NXM1	+
	15	24	6	T4NXM1	+

### Patients characteristics

**Figure 4:**
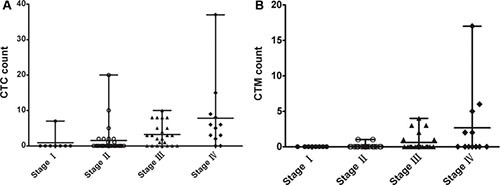
Detection results of CTCs and CTC clusters in different stages of 72 CRC patients

We have detected blood samples from 72 CRC patients and the clinicopathological characteristics were shown in Table 2. There were 35 colon cancer and 37 rectal cancer patients (41 male and 31 female; mean age, 60.2 years; age range, 37–84 years). CTCs were detected in 38 patients with a positive rate of 52.8%. Meanwhile, CTC clusters were found in 13 patients with a positive rate of 18.1%. Detecting results in different stages of patients were shown in Figure 4.

**Table 2:** Clinicopathologic characteristics of 72 CRC patients

Parameter	No. of patients	Percentage (%)
Total	72	
Gender		
male	41	56.9
female	31	43.1
Age		
≤ 60 years	35	48.6
> 60 years	37	51.4
Tumor location		
colon	35	48.6
rectal	37	51.4
Differentiation		
well and moderate	60	83.3
poor	12	16.7
Lymphatic or venous invasion		
No	40	55.6
Yes	32	44.4
Tumor depth		
T1	2	2.8
T2	8	11.1
T3	7	9.7
T4	55	76.4
Lymph node metastasis		
Negative	36	50.0
Positive	36	50.0
TNM stage (UICC)		
Stage I	8	11.1
Stage II	29	40.3
Stage III	23	31.9
Stage IV	12	16.7
CTCs capture		
positive	38	52.8
negative	34	47.2
CTC-clusters		
positive	13	18.1
negative	59	81.9

### Association of CTC detection rate with clinicopathological features

The results were summarized in Table 3. CTC detection rate was associated with the factors of lymphatic or venous invasion, tumor depth, the lymph node status and TNM stage. CRC patients with lymphatic or venous invasion had a higher CTC detecting rate than non lymphatic or venous invasion patients (*p* = 0.001). Moreover, patients with deeper tumor depth had higher CTC positive rate (*p* = 0.008). The CTC positive rate was significantly higher in lymph node metastasis group than no lymph node metastasis group (*p* < 0.001). There was a significant difference of CTC detection rate in different TNM stages (*p* < 0.001). However, there were no significant correlation between CTC detection rate and the factors of age, gender and degrade of differentiation.

**Table 3:** Association of clinicopathological factors with CTCs positive rate

Parameter	No. of patients with CTC (+)	No. of patients with CTC (−)	*P* value
Gender			
male	25	16	0.055
female	13	18	
Age			
≤ 60 years	17	18	0.243
> 60 years	21	16	
Differentiation			
Well and moderate	30	30	0.231
poor	8	4	
Lymphatic or venous invasion			
No	14	26	0.001
Yes	24	8	
Tumor depth			
T1	1	1	0.008
T2	1	7	
T3	1	6	
T4	35	20	
Lymph node metastasis			
Negative	29	7	< 0.001
Positive	9	27	
TNM stage			
Stage I	1	7	< 0.001
Stage II	9	20	
Stage III	17	6	
Stage IV	11	1	

**Figure 5:**
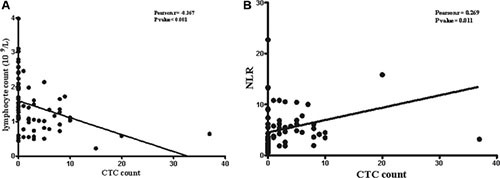
Correlationship of CTC counts with number of lymphocytes and NLR

We have collected the number of blood cells and data of tumor makers, such as carcino embryonie antigen (CEA), carbohydrate antigen-199 (CA199), carbohydrate antigen-125 (CA125) from routine laboratory tests, in order to investigate the correlation with CTCs (Table 4). As for the number of leukocytes, there was no difference between CTC positive and negative groups (*p* = 0.473). Additionally, no significant difference was found in the neutrophil and monocyte counts between the two groups (*p* = 0.245, *p* = 0.491). However, there was significant difference in lymphocyte counts between the two groups (*p* < 0.001). The lymphocyte counts were lower in CTC positive group than negative group and a negative correlation was found between lymphocyte and CTC counts (*p* < 0.001, *r* = **−**0.367, Figure 5A). Moreover, NLR was statistical different between CTC positive and CTC negative group (*p* = 0.024). There was a positive corrlation between NLR and CTC counts (*p* = 0.011, *r* = 0.269, Figure 5B). There were no difference in tumor markers of CEA, CA199 and CA125 between the two groups.

**Table 4:** Analysis of immune cell counts and tumor markers between CTC (+) and CTC (−) group

Parameter	Mean ± SEM CTC (+) *N* = 38	Mean ± SEM CTC (−) *N* = 34	*P* value
Leukocyte count (10^9^/L)	7.19 ± 0.43	7.24 ± 0.58	0.473
Neutrophil count (10^9^/L)	5.66 ± 0.40	5.17 ± 0.59	0.245
Lymphocyte count (10^9^/L)	1.09 ± 0.08	1.85 ± 0.14	< 0.001
Monocyte count (10^9^/L)	0.42 ± 0.05	0.42 ± 0.03	0.491
NLR	6.34 ± 0.85	3.98 ± 0.80	0.024
CEA (ng/ml)	131.10 ± 107.70	3.22 ± 0.42	0.133
CA199 (U/ml)	86.70 ± 40.15	17.77 ± 4.17	0.055
CA125 (U/ml)	25.37 ± 12.27	17.55 ± 2.68	0.278

## DISCUSSION

In our study, we demonstrated a novel ^os^ISET device utilizing a microfabricated membrane filter for CTCs isolation and Wright's staining for CTCs identification. To our knowledge, this is the first time to combine ISET device with Wright's staining as a one-stop automatic device for CTCs detection. In the capture efficiency tests, the device has exhibited a high capture efficiency in detecting either EpCAM positive (MCF-7, A549, SW480), or EpCAM negative cells (Hela), range from 80.3 to 88% in detecting tumor cells from DMEM. Thus, the device showed advantage of capture CTCs no matter they were in epithelial or mesenchymal type. Moreover, compared to IF staining, Wright's staining has been proved to be a feasible and credible way by several researches [27, 28]. In our study, we also found the concordance of the two staining methods, and verified that the Wright's staining was also a feasible method for CTCs identification. Generally, our ^os^ISET device has advantages of detecting CTCs regardless of the expression of specific as well as Wright's staining can identify most CTCs despite their heterogeneity which different from antibody-based isolation and identification methods. Moreover, compared to other size-based isolation systems, like portable filter-based microdevice [20] or SiO2@gel-microbead-based size difference technology [38], the ^os^ISET device have advantages of automation, fast and high-throughput which facilitate its clinical application.

In this study, we also validated the clinical utility of the device to capture CTCs from cancer patients. We have collected samples from 5 breast cancer, 5 lung cancer, 5 gastric cancer and CTCs were detected in 11 patients (3 breast cancer, 4 lung cancer, 4 gastric cancer), CTC-clusters in 5 patients (one breast cancer, 2 lung cancer, 2 gastric cancer). The results revealed the feasibility of ^os^ISET to capture CTCs and CTC-clusters from cancer patients. There was an interesting finding that 8 CTC-clusters were detected in a stage IIA breast cancer while none in metastatic breast cancer patients. This stage IIA breast cancer patient was triple-negative breast cancer which possessed a high potential of distant metastasis. We hypothesized the CTC-clusters presence was associated with molecular subtype of breast cancer not only TNM stage. Maheswaran et al. have verified that CTC-clusters arised from oligoclonal tumor cell groupings and possessed 23–50-fold increased metastatic potential [39]. CTC-clusters have been considered as a key role in initiating distant metastasis and has been validated by Toner et al. [40] that it also can traverse capillary-sized vessels under physiological conditions due to their ability to rapidly and reversibly unfold into single-file chains by cleavage of intercellular adhesions. It has been revealed that in breast cancer patients, high abundance of CTC-clusters had worse outcomes. Thus, the detection of CTC-clusters in cancer patient provides more important information for cancer management which is a distinct advantage of ISET device than epithelial marker-based devices. Finally, in detection samples of 72 CRC patients, CTC positive rate was 52.8% and CTC-clusters positive rate was 18.1%. In control group, no CTCs were detected by our device. Thus the device exhibited a high efficiency in capture CTCs and CTC-clusters.

Futhermore, we have explored the association of CTC positive rates with clinicopathological features of CRC. In this study, we found that there were several factors associatied with CTC positive rates, including lymphatic or venous invasion, tumor depth, lymph node status and TNM stage. No significant difference were found in factors of age, gender and degrade of differentiation. From our experimental data, we found CTC positive rates in local advanced and distant metastasis patients were significantly higher than early stage patients (*p* = 0.008). Patients with lymphatic or venous invasion, deeper tumor depth or lymph node metastasis had a higher CTC detection rates (*p* < 0.01). Our result has revealed that CTC positive rates were associated with indicators of poor prognosis. Therefore, CTCs may be an important prognostic indicator in CRC which has been validated by other research that its presence associated with shorter DFS and OS [30–32]. A recent study showed that both CTC detection rates and CTC counts had significant correlation with tumor progression and the appearance of distant metastases in non-metastatic CRC patients [32].

Although millions of tumor cells shed from primary tumor into the blood stream every day, only few can be detected at any given time [41]. We hypothesize that CTCs as one component of blood cells, the counts may not only relate to primary tumor, but also are influenced by other cells in blood circulation, such as clearance by immune cells. So we have explored the association of immune cell number and neutrophil-to-lymphocyte ratio (NLR) with CTC counts. We found that the number of lymphocytes in CTC positive group was less than CTC negative group. But there was no difference in neutrophil and monocyte counts. Moreover, NLR was lower in CTC positive group than CTC negative group. There was a negative correlation between lymphocyte and CTC counts (*p* < 0.001, *r* = −0.367) while a positive correlation between NLR and CTC counts (*p* = 0.011, *r* = 0.269). Based on this results, we concluded that CTC counts were associated with number of lymphocytes not neutrophils and monocytes. Thus, lymphocytes may play a main role in the clearance of CTCs from the blood stream. It has been reported that natural killer cells, as one type of lymphocytes, play a key role in the antitumor immunity [42]. Recent study has revealed the mechanism of immune escape of CTCs in CRC patients that CTCs exhibited a distinct nonimmunogenic phenotype by overexpressing CD47 which was the only gene found significantly upregulated. And CD47 is a protein inhibiting the cytotoxic and phagocytic activity of T cells and macrophages which is associated with tumor cell immune escape [43]. Futhermore, some studies have revealed the association of CTC number with immunity in cancer patients. Patients with higher positive CTC number than the baseline had decreased immune function in comparison to those lower than baseline or negative detection, especially for the patient with distant metastasis [44]. In other studies, higher level of CTC detected in breast cancer with bone metastases were correlated with lymphocytopenia [45, 46]. In fact, immune responses reduce reactivity of disseminated tumor cells, which reflect in the number of CTCs and lymphocytes [47]. Moreover, the number of CTCs and lymphocytes not only revealed their interaction effect but also were reported to be prognostic indicators in cancer patients. A recent study about metastatic breast cancer showed that low lymphocyte and high CTC counts were independent poor predictive and prognostic factors [48]. Additionally, NLR was reported to associate with prognosis of colorectal cancer [49]. NLR< 5 was predictor of shorter DFS and OS in CRC [50]. Therefore, number of lymphocytes and CTC counts may be indicators to estimate immune condition, guide immunotherapy and predict prognosis.

Also, there are some limitations of our study. First of all, the purity of CTCs need to be concerned. We found that the percentage of blood cells to CTCs was still relatively high. However, this did not affect the identification of CTC. Second, our ^os^ISET device is still hard to release CTCs from the membrane. This may limit the application of downstream genetic analysis. Laser capture microdessection may solve this problem. In our study, the number of clinical cases was small and our results were needed to interpret with caution.

In conclusion, our ^os^ISET device realized the one-stop capture and identification for CTCs and CTC clusters. Based on the results of our study, we have validated the feasibility of ^os^ISET device to detect CTCs and CTC clusters from cancer patients. The more important is that through association research of CTCs with clinicopathological features in CRC patients, indicated that detection CTCs by our ^os^ISET device may play a role in therapeutic effect evaluation and prognostic prediction.

## MATERIALS AND METHODS

### Cell culture

MCF-7 (human breast cancer cells), A549 (human lung cancer cell), SW480 (human colonal cancer cell), Hela (human cervical caner cell) were obtained from China Center for Type Culture Collection (CCTCC). Cells were cultured in Dulbecco's modified eagle medium (DMEM, Hylcone, Thermo scientific, USA) added with 10% fetal bovine serum (sigma, USA) and 1% penicillin/streptomycin at 37°C in 5% humidified CO_2_ incubator.

### Capture efficiency tests

MCF-7, A549, SW480, Hela cells were spiked into DMEM medium or blood sample at concentrations of 100 cells per 2.5 mL. SW480 cells were spiked into DMEM medium or blood sample at a series concentrations of 50, 100, 150, 200 cells per 2.5 mL. The spiked samples were detected by the ^os^ISET device through whole procedure. Each test was repeated three times.

### Isolation and staining procedure of ^os^ISET device

**Figure 6:**
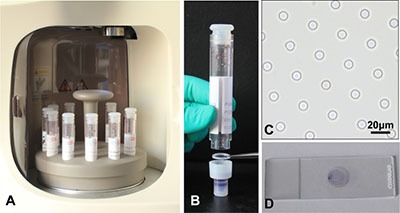
Introduction of the osISET device (**A**) The blood samples were waiting for isolation. (**B**) The filter membrane picked by the forceps was at the bottom of a cylindrical filtration. (**C**) The calibrated 8-μm-diameter pores were shown. (**D**) The transparent filter membrane was placed on slide after automated isolation.

Blood sample (2.5 ml) was diluted 1:2 with the BD wash buffer (BD, USA) containing 0.2% paraformaldehyde (PFA), 0.1% bovine serum albumin (BSA), and 0.0372% EDTA. Left it for 10 minutes at room temperature and then detected by the device. The device has 10 wells, making it possible to load and filter 10 individual samples in parallel (Figure 6A). The filteration was gently aspirated by the vacuum suction pump. After aspiration, the retained cells were washed three times by pure water and fixed by 100% methanol. Then cells were stained with eosin and followed by methylene blue, and washed with PBS through a multitendam valve. Then the whole procedure was completed which only takes 10 minutes for each sample. Disassembled from the filtration (Figure 6B), the membrane (Figure 6C) was placed on a slide and coverslipped after air-dried (Figure 6D).

### IF staining and subsequent Wright's staining

We performed IF staining and Wright's staining on the same sample to test the concordance of the two staining method in identification CTCs. The spiked sample and clinical samples were used. After isolation, the filter membrane were taken out from the device for subsequent IF staining and Wright's staining. Captured cells on the membrane were fixed with 4% PFA for 5 minutes. Wash the membrane by BD wash buffer (BD, USA) for three times. Then, add 100 ul Cytofix/Permeabilization Kit (BD, USA) on the membrane for 20 minutes in order to allow for intracellular staining. After that, add 10% Goat serum to block for one hour. Then, discard the serum and add the primary mouse antibody to pan-CK (Abcam, USA) and rat antibody to CD45 (Santa, USA) diluted 1:100 for incubation overnight at 4°C. On the next day, wash the membrane by BD wash buffer and add the secondary Alexa Fluor 488-conjugated goat anti-mouse IgG (Invitrogen, USA) and Alexa Fluor 594-conjugated goat anti-rat IgG (Invitrogen, USA) diluted 1:200. Nuclei was stained with Hoechst 33342 (Sigma, USA) diluted 1:500 and incubated for one hour. At last, wash the membrane and observe on the fluorescence microscopy. CK+/CD45−/Hoechst+ cell was scored as CTCs and CK−/CD45+/Hoechst+ cell as white blood cell (WBC). Subsequent wright's staining was performed as follows: slides were immersed in 100% xylene for several minutes at room temperature until the cover glasses dropped off. Then, add eosin on the membrane for 2 minutes and then discard. Next, add methylene blue for 1 minutes, and then washed with PBS. Then the membrane was air-dried and observed on light microscopy. The results of IF staining and Wright's staining were blindly reviewed by two group independently. The criterion for identification CTCs and CTC clusters confirm to the cytomorphological criteria proposed by other research groups [27, 28]. The result of Wright's staining was identified by two experienced cytopathologists.

### Detecting blood samples of cancer patients and healthy donors

Whole blood samples from healthy donors were obtained from the department of health examination center, Zhongnan Hospital of Wuhan University according to Institutional Review Board (IRB) protocol. Blood samples from cancer patients, including breast cancer, lung cancer, gastric cancer, colorectal cancer were obtained from the department of surgical oncology, Zhongnan Hospital of Wuhan University from January to June 2016. Peripheral blood samples (2.5 ml) were collected on EDTA buffer and processed by the device through the automatic isolation and staining procedure. All the samples were collected before initial treatment and handled within 4 hours. All the participants have provided their written informed consent to participate in this study. This research was approved by the Medical Ethical Committee of Zhongnan Hospital.

### Data collection

Clinicopathologic data of patients were collected from hospital information system, including age, gender, tumor location, differentiation degree, tumor depth, lymph node status, TNM stage information, results of blood routine tests and tumor markers. The pathologic stages were confirmed to the guidelines of National Comprehensive Cancer Network of America (NCCN).

### Statistical analysis

All statistical calculations were performed with Statistical Package for Social Science (SPSS version 19.0). The χ^2^ analysis or Fisher exact test were used to explore correlation between CTCs detecting rate and patients’ clinicopathological characteristics. Student's *t*-test was used to compare immune cell number, tumor marker level, NLR between CTC positive and negative groups. Correlation analysis and regression were made by Pearson correlation analysis. Differences were considered statistically significant when the *p* value was < 0.05.
